# Superconducting Fullerene Nanowhiskers

**DOI:** 10.3390/molecules17054851

**Published:** 2012-04-26

**Authors:** Hiroyuki Takeya, Kun’ichi Miyazawa, Ryoei Kato, Takatsugu Wakahara, Toshinori Ozaki, Hiroyuki Okazaki, Takahide Yamaguchi, Yoshihiko Takano

**Affiliations:** 1 National Institute for Materials Science, Tsukuba, Ibaraki 305-0047, Japan; 2 Transformative Research Project on Iron Pnictides (TRIP), Japan Science and Technology Agency, Chiyoda-ku, Tokyo 102-0075, Japan

**Keywords:** fullerene nanowhisker, superconductor, potassium intercalation

## Abstract

We synthesized superconducting fullerene nanowhiskers (C_60_NWs) by potassium (K) intercalation. They showed large superconducting volume fractions, as high as 80%. The superconducting transition temperature at 17 K was independent of the K content (x) in the range between 1.6 and 6.0 in K-doped C_60_ nanowhiskers (K_x_C_60_NWs), while the superconducting volume fractions changed with x. The highest shielding fraction of a full shielding volume was observed in the material of K_3.3_C_60_NW by heating at 200 °C. On the other hand, that of a K-doped fullerene (K-C_60_) crystal was less than 1%. We report the superconducting behaviors of our newly synthesized K_x_C_60_NWs in comparison to those of K_x_C_60_ crystals, which show superconductivity at 19 K in K_3_C_60_. The lattice structures are also discussed, based on the x-ray diffraction (XRD) analyses.

## 1. Introduction

Fullerenes were discovered in 1985 [1], and the superconductivity of a potassium metal-doped fullerene was reported in 1991 [2]. Potassium metal (K)-doped fullerides K_x_C_60_ [0 ≤ x ≤ 6] are particularly interesting because their structures and electronic properties are strongly related to the doping concentration. They exhibit an fcc structure at x = 0 and 3, a bct structure at x = 4, and a bcc structure at x = 6 [3]. The compound, K_3_C_60_, shows superconductivity below 19 K, while the others exhibit insulating, semiconducting or metallic properties. In general, superconducting K_3_C_60_ bulk samples have been synthesized mainly by three methods, *i.e.*, a solid-solid reaction, vapor evaporation, and a reaction using liquids. Much effort has been expended to produce the K_3_C_60_ superconductor, but a large volume fraction was difficult to obtain by a simple heating method. In addition, the obtained bulk superconductors of K_3_C_60_ by the above methods were usually in powder form. The above two were the problems inherent in the bulk application of K_3_C_60_ superconductors. 

A recent study of fullerene-based supramolecular nanoarchitectures [4,5,6,7,8,9] is opening new possibilities for the application of fullerene materials; such applications include sensors, transistors, catalysts, and fuel cell electrodes. Fullerene nanowhiskers were obtained from the interface between the fullerene-saturated solution and fullerene-insoluble solvent. They vary in length from microns to centimeters. Most of the cuprate superconductors form powders or bulk polycrystals, which were encapsulated in metal tubes for making superconducting wires. This makes the process complicated and the wire heavy. In this study, we propose fullerene nanowhiskers for use as flexible and lightweight superconducting wire because of the advantages in the nanowhisker form. 

## 2. Results and Discussion

### 2.1. Observation of Morphologies

**Figure 1:**
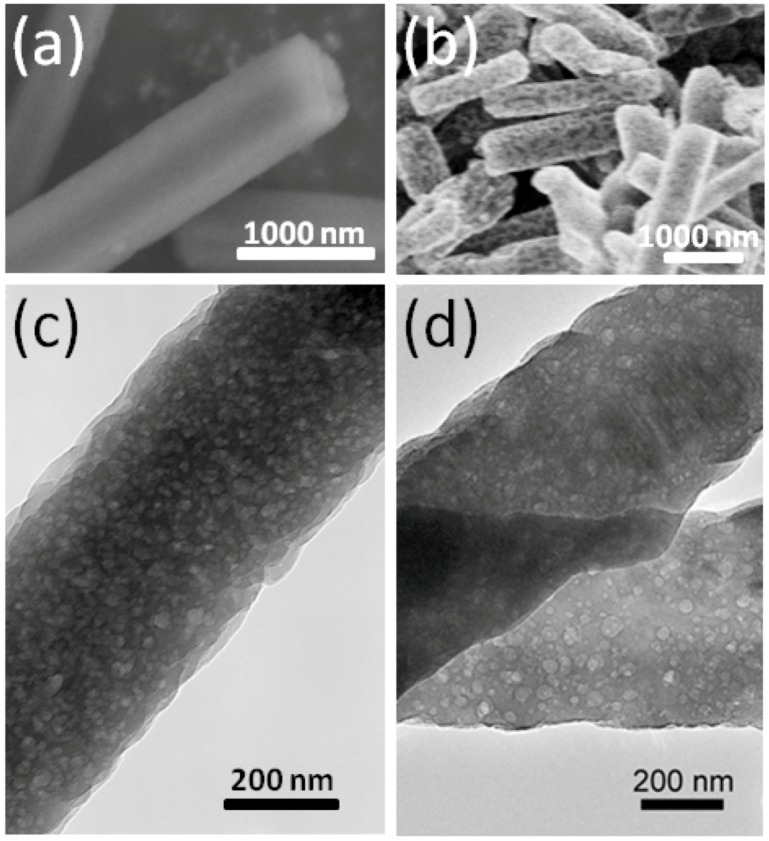
SEM micrographs of (**a**) C_60_NWs and (**b**) K-doped C_60_NWs. (**c**) and (**d**) are the micrographs of (**a**) and (**b**) observed using a transmission electron microscope (TEM), respectively.

The morphology of C_60_ nanowihskers (C_60_NWs) used in this study are shown in Figure 1(a) observed using a scanning electron microscope (SEM). The shapes of C_60_NWs are mostly hexagonal prisms with the growth axis along the [001] direction [10]. 

C_60_NWs grow in a hexagonal crystal structure by the liquid-liquid interfacial method (LLIP method) [4,5], and the hexagonal structure then turns to the fcc structure while the solvent is drying. The C_60_NWs are composed of C_60_ units bound by van der Waals force. Figure 1(c) shows the micrograph of C_60_NWs observed using a transmission microscope (TEM), indicating disordered nanopores formed at the stage of the drying process in the LLIP method. The 10 mg C_60_NWs were taken in a quartz tube with an appropriate amount of K (molar ratio K/C_60_ = 0–6.0). The quartz tube was then sealed under an evacuated condition at about 3 × 10^−3^ Pa and heated at 200 °C for 24–36 h for K intercalation into C_60_NWs. The SEM micrograph and TEM image of the K-intercalated samples of K_3.3_C_60_NWs are shown in Figure 1(b) and 1(d), respectively. Basically, the morphology of the K-doped samples looks the same as the pristine samples showing the nanopore structure. The energy dispersive x-ray analysis (EDAX) revealed that K was taken in the C_60_NWs. In this report, all of the compositions denote nominal ones. We also prepared K-doped C_60_ crystals to compare the K-doped C_60_NWs. Several micro-cracks were observed in K_3.3_C_60_ crystals, while no crack was observed in K_3.3_C_60_NWs. K-doping into the C_60_ crystal expands the lattice of the C_60_ crystals; therefore, cracks seemed to be induced because of the boundary strain from the lattice mismatch between K-doped and undoped lattices. On the other hand, in the case of K-doped C_60_NWs, the disordered nanopores play a possible role in reducing the lattice strain.

### 2.2. Superconducting Properties and X-ray Diffraction Patterns

**Figure 2:**
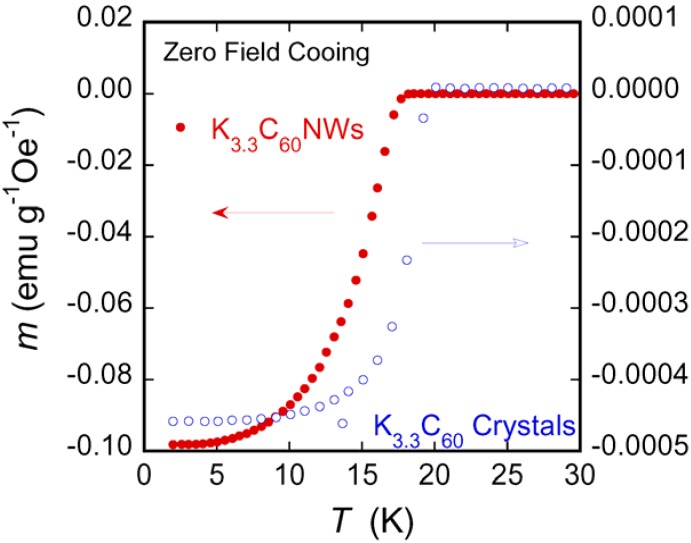
The temperature dependencies of the normalized magnetic moments *m* for K_3.3_C_60_NWs and K_3.3_C_60_ samples.

Figure 2 shows the superconducting transitions of K_3.3_C_60_NWs and K_3.3_C_60_ crystals heated at 200 °C for 24 h, which were measured upon warming under 20 Oe after cooling in a zero-field. The nominal K composition indicates the ratio *vs.* the C_60_ unit.

The left and right ordinates of the graph set separately for K_3.3_C_60_NWs and K_3.3_C_60_ crystals are the magnetic moment normalized by the applied magnetic field and the weight of the samples. In K-doped C_60_ materials, the only fcc phase of stoichiometric K_3_C_60_ is a superconductor. We added a 10% excess amount of K, more than the crystallographically limiting composition K_3_C_60_NW and K_3_C_60_, because the K adsorption on the surface of crystallites or in the nanopores was considered to ensure complete reaction. As shown in the figure, there is a large difference of the superconducting signals between the two samples. The signal of the nanowhisker sample was 200 times larger than that of the crystal sample. The *T*_c_ at 17 K in K_3.3_C_60_NWs is lower than the reported *T*_c_ at 19 K in K_3.3_C_60_ crystals, as shown in Figure 2. We believe the *T*_c _reduction in K_3.3_C_60_ NWs can be caused from the disordered nanopores as seen in Figure 1(d).

**Figure 3:**
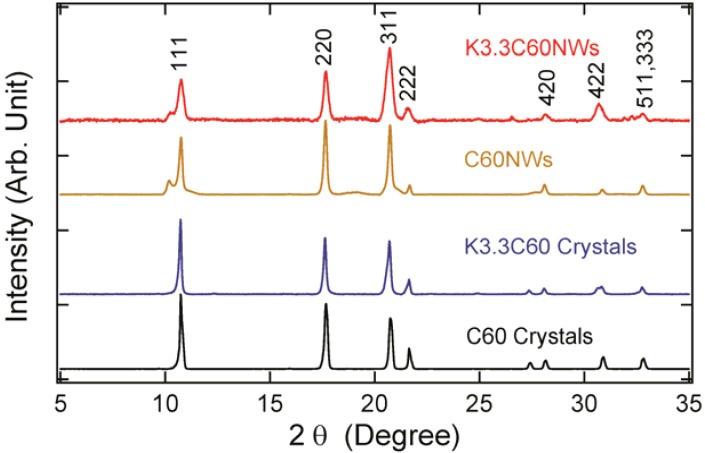
XRD patterns of C_60_ crystals, K_3.3_C60 crystals, C_60_NWs, and K_3.3_C_60_NWs (nominal composition), with fcc structure index.

**Figure 4:**
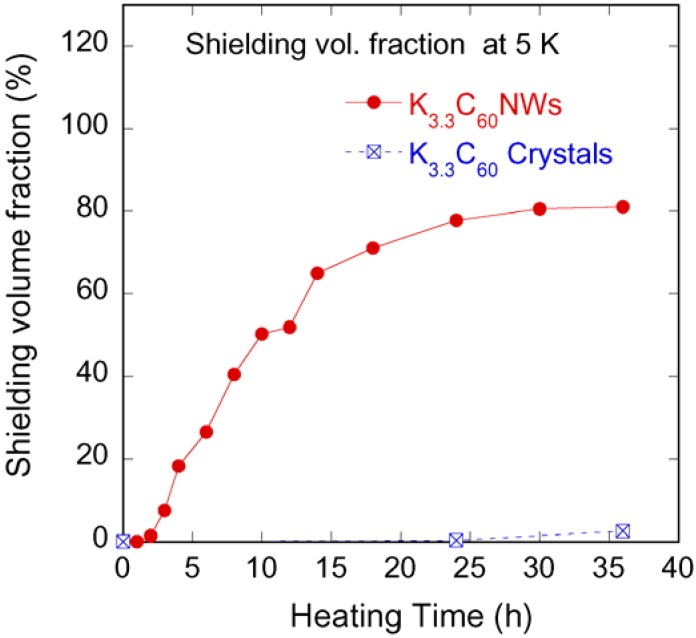
Formation of superconducting phase (%) in K_3.3_C_60_NWs estimated by shielding volume fractions *vs.* heating time (h) at 200 °C.

The powder x-ray diffraction (XRD) patterns of C_60_, K_3.3_C_60_ crystals, C_60_NWs, and K_3.3_C_60_NWs are shown in Figure 3. The XRD patterns of non-doped C_60_ crystals and C_60_NWs showed an fcc structure, as previously reported [3]. In addition, those of K_3.3_C_60_ crystals and K_3.3_C_60_NWs were also identified to be the fcc structure phase as the peaks were indexed in the figure. The lattice parameters of four samples, C_60_, K_3.3_C_60_ crystals, C_60_NWs, and K_3.3_C_60_NWs, were calculated to be 1.4180(5), 1.4180(2), 14.188(4), and 1.4200(5) nm, respectively. Therefore, K was hardly intercalated into the sites in the K_3.3_C_60_ crystals because its lattice parameter was almost identical to that of C_60_ crystals. This result is consistent with the difference of the superconducting shielding volume fractions between K_3.3_C_60_NWs and K_3.3_C_60_ crystals by the nominal compositions. According to reported processes for K-doping to C_60_, so far, a period longer than several days or several weeks is required to form the superconducting K_3_C_60_ phase [11,12]. We investigated the formation rate of the superconducting phase by measuring the superconducting volume fractions at 5 K as shown in Figure 4. In the case of K_3.3_C_60_NWs, it was saturated at around 24 h with heating at 200 °C. On the other hand, the shielding fraction of K_3.3_C_60_ crystals hardly increased up to 36 h. Such a small volume fraction of the superconducting K_x_C_60_ has already been reported, for example, by Hebard *et al.* [2] and Murphy *et al.* [3] Hebard *et al.* reported approximately 1% by heating at 200 °C for 36 h. We believe that the nanopores of K_3.3_C_60_NWs, shown in Figure 1(d), assist in the reaction of K-doping and the migration to form the K_3_C_60_NW superconducting phase, as explained in Figure 2. The nanopores in K_3.3_C_60_NWs play an important role for the formation of superconducting phase.

### 2.3. K-Compositional Dependence of Shielding Volume Fraction in K_x_C_60_NWs

**Figure 5:**
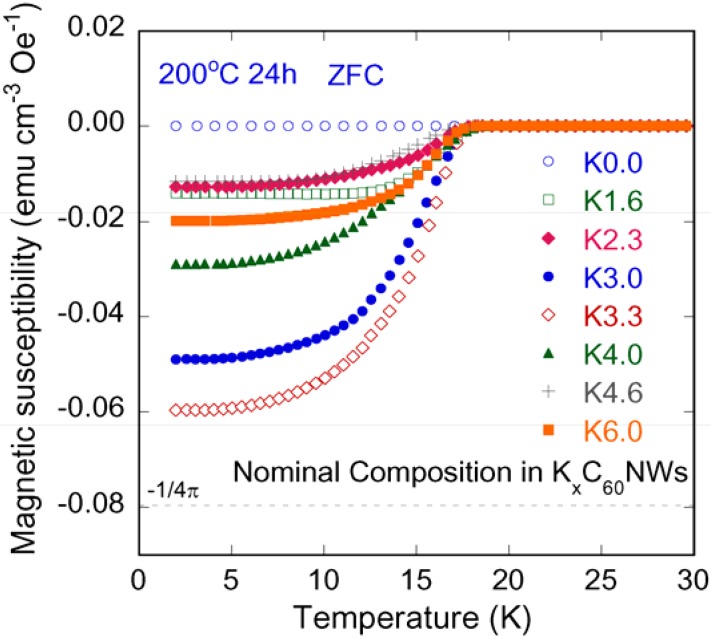
The temperature dependencies of magnetic susceptibility for K_3.3_C_60_NWs (x = 0.0–6.0).

Figure 5 illustrates the bulk superconducting transitions of K-doped C_60_NWs for the K composition range of x = 0.0–6.0, which were measured on warming under 20 Oe after cooling in a zero-field. 

The K composition indicates the nominal ratio *vs.* C_60_ unit in the C_60_NWs. The ordinate of the graph is the magnetic moment normalized by the applied magnetic field and the volume of the samples. None of the samples before heating showed any anomalies within a temperature range between 2 K and 30 K. The onset *T*_c_’s of the materials after heat treatment at 200 °C were almost the same (17 K) independently of the K composition, while the superconducting shielding fractions depended on the K-ratio.

**Figure 6:**
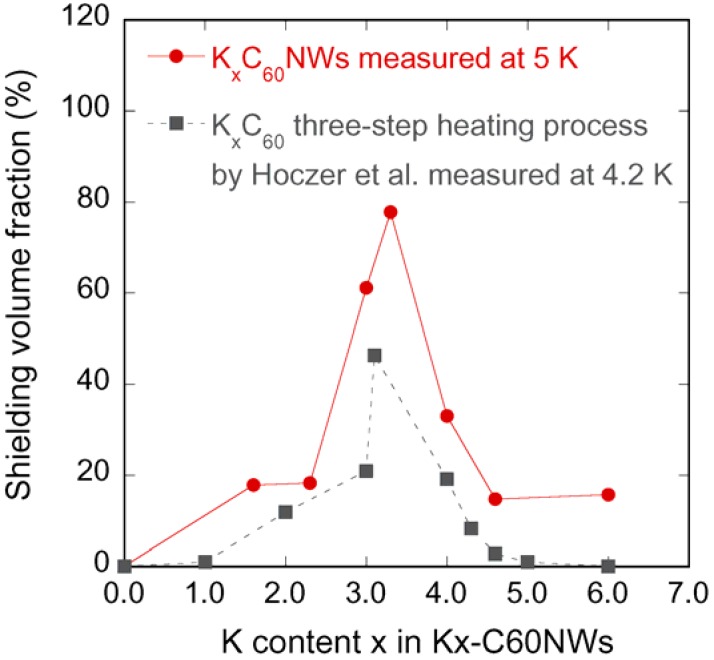
K content dependence of shielding volume fractions in K_x_C_60_NWs (at 5 K) compared with the result by Hoczer *et al.* [11] for K_x_C_60_ (at 4.2 K).

Figure 6 shows the compositional dependence of the superconducting volume fractions in K_x_C_60_NWs (nominal composition) heated at 200 °C for 24 h in comparison with those in K_x_C_60_ reported by Holczer *et al.* [11]. 

Their heating procedure consists of three stages which are: (1) the first mixing stage heated at 200 °C for 20–24 h, (2) the second diffusion stage heated to 200 °C for 22 h and (3) the final relaxation stage heated at 250 °C for 6 h or more hours. Basically, their result coincides with our result in K_x_C_60_NWs. The shielding volume fractions are normalized by the volume of the perfect diamagnetism (−1/4π). The maximum fraction was observed at around 3.0–3.3 by the nominal K compositions. This value coincides with the carrier concentration at the *T*_c_ maximum in alkali-doped C_60_ superconductors [12]. In the K_x_C_60_ system, the superconducting phase is a line compound of K_3_C_60_ with the full occupancy of two tetrahedral and one octahedral sites by K in the fcc structure. The three electrons transferred from K to C_60_ occupy the triply degenerated *t*_1u_ orbital, which becomes half-filled, and the high density of states at the Fermi level. We believe that this logic of K_3_C_60_ is analogical for K_3_C_60_NW. On the other hand, there is a difference in the case of x = 6.0 in K_x_C_60_NW, showing some superconducting volume fraction. Murphy *et al.* explained that the non-superconducting bcc phase (K_6_C_60_) was formed as a kinetically facile phase at the first step of the reaction [13]. Regarding our XRD measurement for the nominal K_6_C_60_NW in the heating condition at 200 °C by 36 h, no bcc phase was detected in contrast to the Murphy’s explanation. Since the lattice parameter of K_6.0_C_60_NW is 1.4210(9) nm, which is almost the same as 1.4200(5) nm of K_3.3_C_60_NW, an excessive amount of K over 3.0 might stay at the surface or in the nanopores of C_60_NW. 

## 3. Experimental

**Figure 7:**
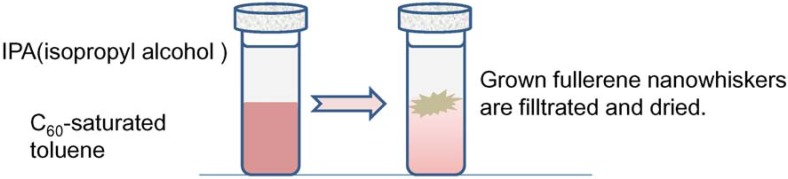
A schematic diagram of the liquid-liquid interfacial method (LLIP method).

The typical dimensions of the fullerene nanowhiskers (C_60_NWs) used in this experiment were 0.54 ± 0.16 μm in average diameter and 4.43 ± 2.63 μm in average length. The C_60_NWs were prepared by using the liquid-liquid interfacial method (LLIP method) [4,5]. The schematic diagram is illustrated in Figure 7. A C_60_-saturated toluene solution was taken in a glass bottle, and isopropyl alcohol was slowly added. The C_60_NWs form at the interface of the two solutions, then the nanowhiskers were filtered and dried in vacuum at 100 °C for 2 h [14]. According to the previous report [15], the residual toluene solvent in C_60_NWs is estimated to be about 0.2 mass %.

Ten mg C_60_NWs and an appropriate amount of potassium (K) were placed together into a thin quartz tube. The nominal K compositions were set at 0.0, 1.6, 2.3, 3.0, 3.3, 4.0, 4.6 and 6.0 mole ratio *vs.* C_60_ in K_x_C_60_NWs. We also prepared pristine and K-doped C_60_ crystals using the same procedures for a comparison with K_x_C_60_NWs. This process was conducted in a glove box to prevent potassium from oxidizing. The quartz tube was sealed under a vacuum condition at 3 × 10^−3^ Pa, followed by heating at 200 °C for 1–36 h in an electric oven. After the heat treatment, superconducting properties and structure analyses were performed as follows. Superconducting transitions were measured using a superconducting quantum interference device (SQUID) magnetometer (MPMS-5S, Quantum Design, San Diego, CA, USA) as the samples were kept in the quartz tube. The shapes and microstructures of those samples were observed with a scanning electron microscope (SEM 25kV, Hitachi SU-70, Tokyo, Japan) and a transmission electron microscope (TEM 400kV, JEOL JEM-4010, Tokyo, Japan). Their qualitative micro-analysis was achieved with an energy dispersive X-ray analyzer (EDAX, AMETEK, Mahwah, USA). The information of the crystal structure was analyzed by powder X-ray diffraction (XRD RINT-TTR3, RIGAKU, Akishima, Japan). To prevent the XRD samples from being oxidized, we used a tiny amount of paraffin oil to cover them on the holder plate. In the XRD patterns, the diffraction of oil was subtracted from the raw data.

## 4. Conclusions

We proved that K intercalation to C_60_NWs, rather than the process for K_3_C_60_, forms superconducting K_3_C_60_NWs with a short heating process. No bcc phase of K_6_C_60_NW was observed. The superconducting shielding volume fraction by heating at 200 °C for 24 h gave high values up to 80%, in contrast to the value of K-doped C_60_, K_3.3_C_60_, which was lower than 1%. This contrasting difference of the superconducting shielding fraction might be associated with the nanopores in K_x_C_60_NWs. These nanopores play an important role in the properties of K_x_C_60_NWs.
